# Supplementary material for: Klotho deficiency intensifies hypoxia-induced expression of IFN-α/β through upregulation of RIG-I in kidneys
Source: PLoS One. 2021 Oct 21;16(10):e0258856. doi: 10.1371/journal.pone.0258856 (PMC8530307; doi:10.1371/journal.pone.0258856)
Supplement: S1 Fig — (PDF) [file pone.0258856.s001.pdf]

Supporting Information

Klotho deficiency intensifies hypoxia-induced expression of IFN-α/β through upregulation of RIG-I in kidneys

Asako Urabe[1], Shigehiro Doi[1]*, Ayumu Nakashima[1,2], Takeshi Ike[1], Kenichi Morii[1], Kensuke Sasaki[1], Toshiki Doi[1], Koji Arihiro[3], Takao Masaki[1]*

[1]Department of Nephrology, Hiroshima University Hospital, Hiroshima, Japan; [2]Department of Stem Cell Biology and Medicine, Graduate School of Biomedical & Health Sciences, Hiroshima University, Hiroshima, Japan; [3]Department of Anatomical Pathology, Hiroshima University Hospital, Hiroshima, Japan

S1 Fig.

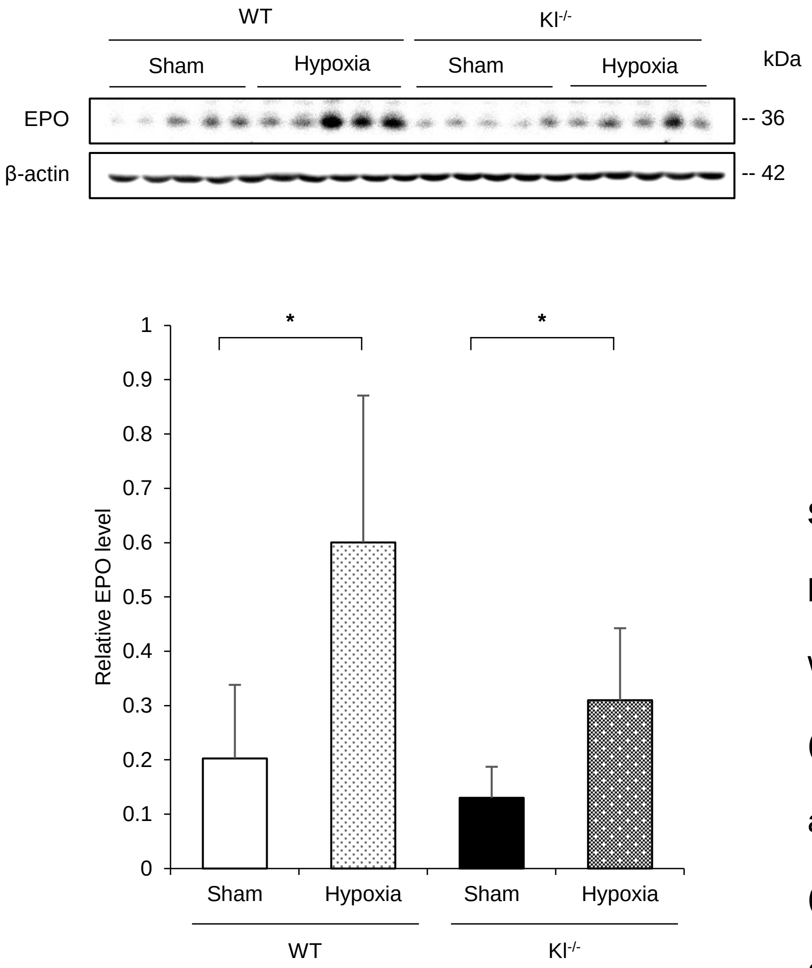

S1 Fig. Erythropoietin expression is intensified under hypoxic conditions in WT and Kl$^{-/-}$ mice

Western blot analysis demonstrating erythropoietin (EPO) (66975-1-Ig; proteintech, Rosemont, IL, USA) expression in WT and Kl$^{-/-}$ mice. Protein levels were normalized to β-actin levels (n=5 in each group). Values are mean ± SD. *P < 0.05, **P < 0.01.
