# Supplementary material for: Klotho deficiency intensifies hypoxia-induced expression of IFN-α/β through upregulation of RIG-I in kidneys
Source: PLoS One. 2021 Oct 21;16(10):e0258856. doi: 10.1371/journal.pone.0258856 (PMC8530307; doi:10.1371/journal.pone.0258856)
Supplement: S2 Fig — (PDF) [file pone.0258856.s002.pdf]

S2 Fig.

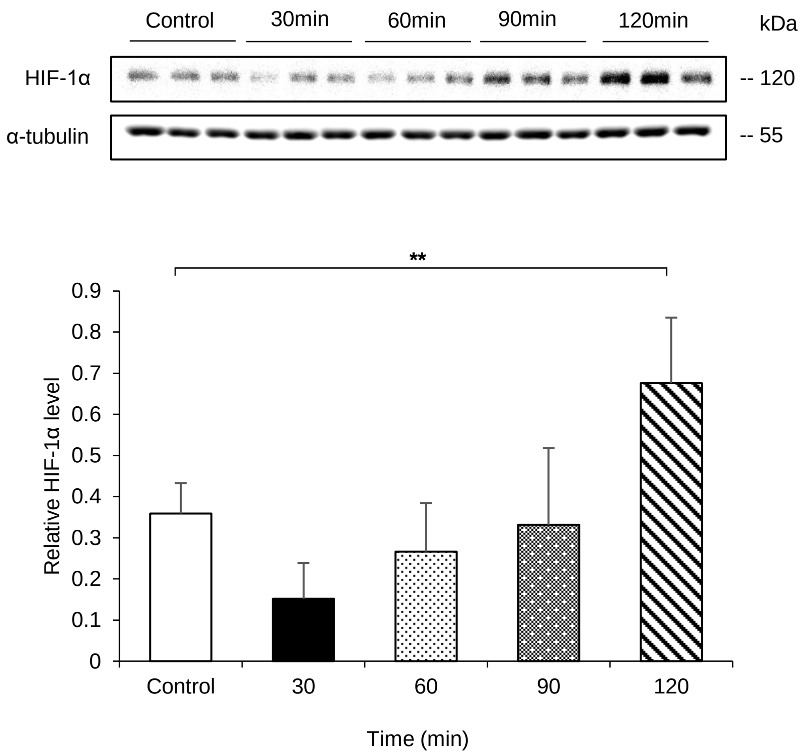

S2 Fig. Hypoxia enhances expression of HIF-1α in a rat cell line of epithelial cells of renal tubules

NRK-52E cells were incubated under hypoxic conditions (1.0% O_2) for 30, 60, 90, and 120 min. Cell lysates were subjected to western blot analysis using antibodies against HIF (Hypoxia Inducible Factor)-1α (#36169S; Cell Signaling Technology, Danvers, MA, USA). Typical western blot analysis demonstrated the expression levels of HIF-1α. Graphs show the expression levels quantified by densitometry and normalized to α-tubulin (n=5 in each group). Values are expressed as the mean ± SD. Statistical analysis was performed using ANOVA followed by Tukey's post hoc test. **P* < 0.05, ***P* < 0.01.
