# Supplementary figures and images for: Klotho deficiency intensifies hypoxia-induced expression of IFN-α/β through upregulation of RIG-I in kidneys
Source: PLoS One. 2021 Oct 21;16(10):e0258856. doi: 10.1371/journal.pone.0258856 (PMC8530307; doi:10.1371/journal.pone.0258856)

S4 Fig. raw images of western blots for Fig 1A.

Fig 1A

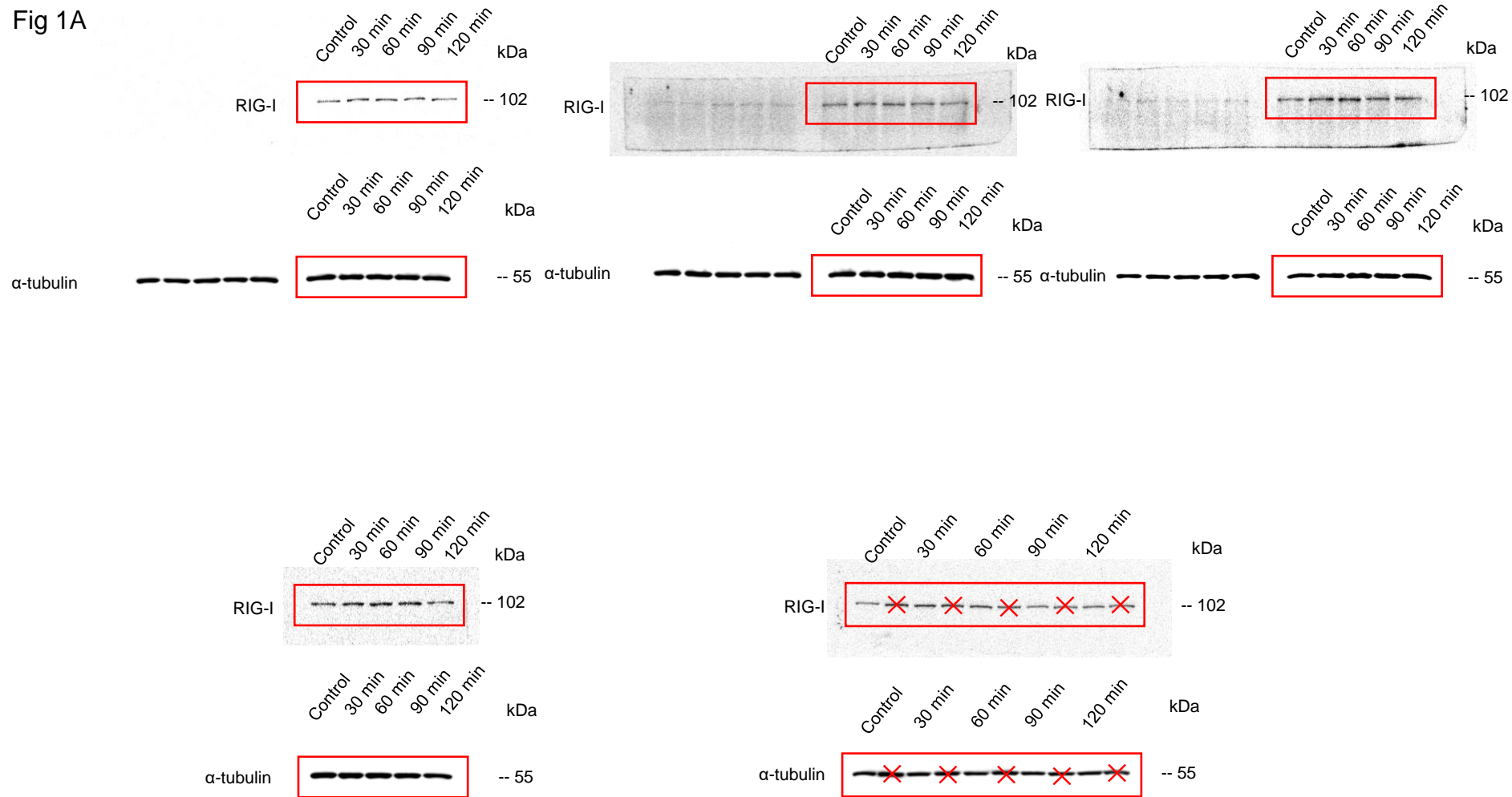

Supplement: S3 Fig — (PDF) [file pone.0258856.s003.pdf]

S5 Fig. raw images of western blots for Fig 1B.

Fig 1B

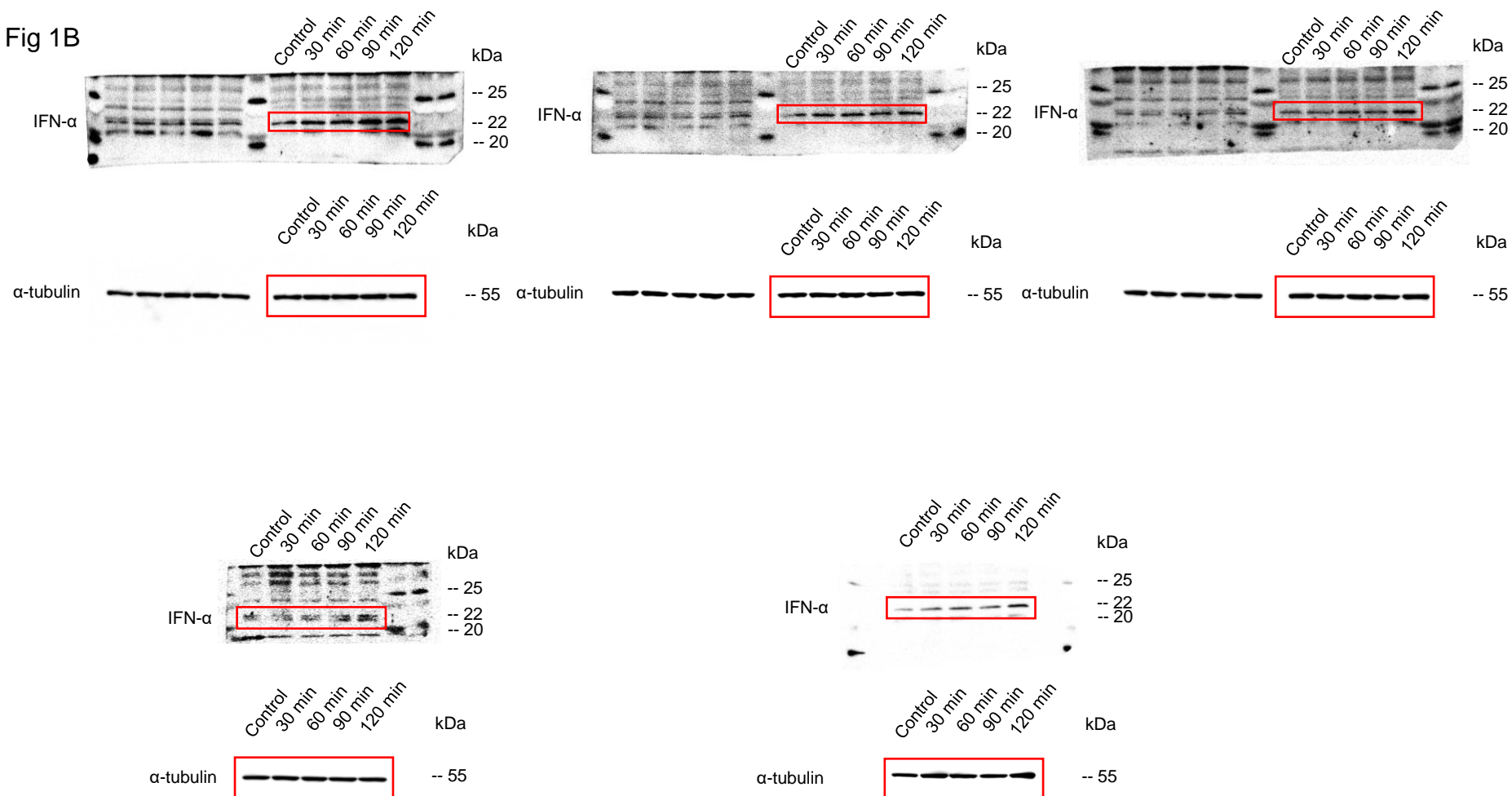

Supplement: S4 Fig — (PDF) [file pone.0258856.s004.pdf]

S6 Fig. raw images of western blots for Fig 1C.

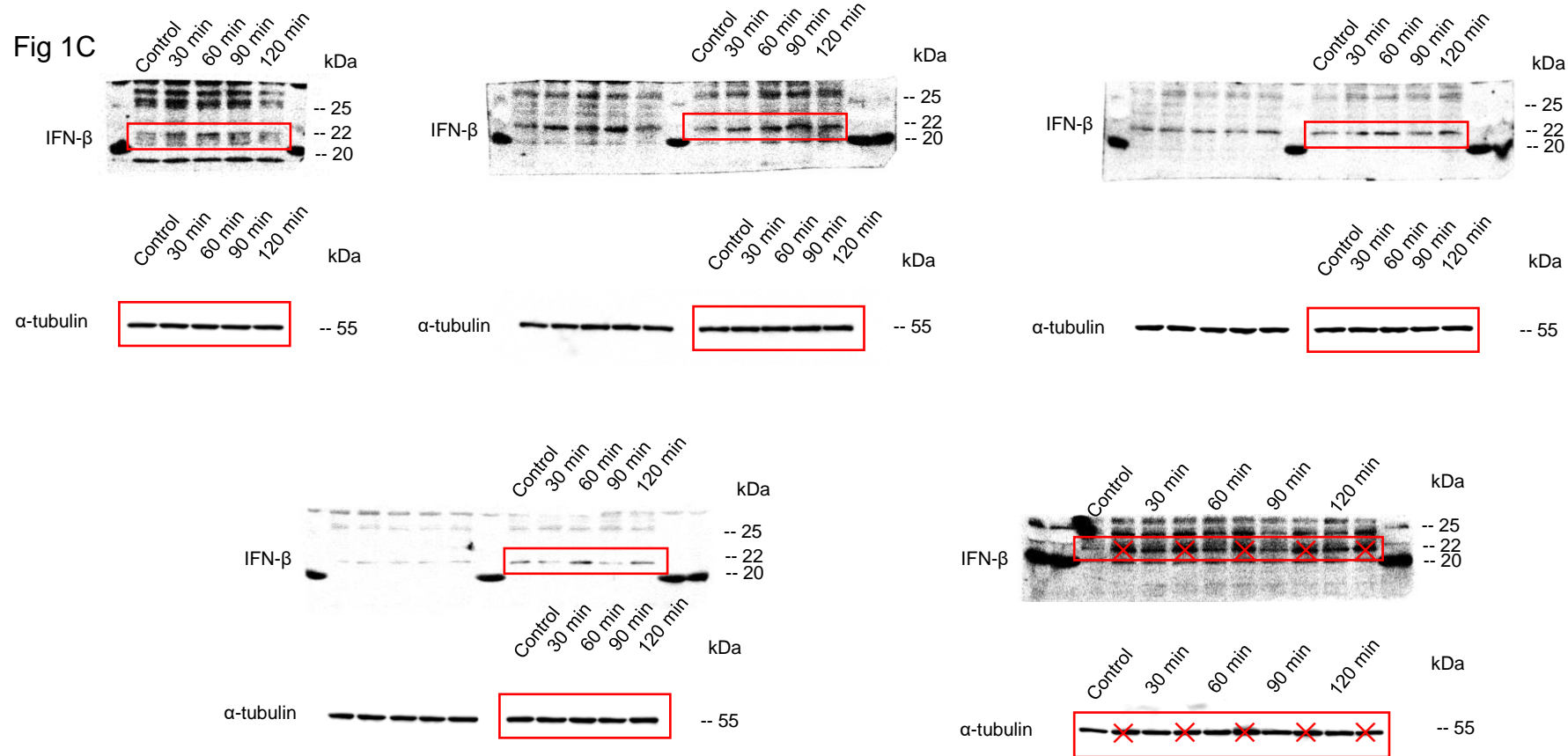

Supplement: S5 Fig — (PDF) [file pone.0258856.s005.pdf]

S7 Fig. raw images of western blots for Fig 2A, 2B, and 2C.

Fig 2A

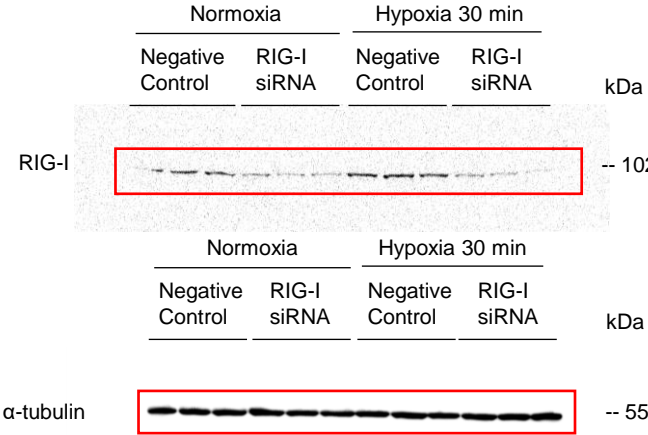

Fig 2B

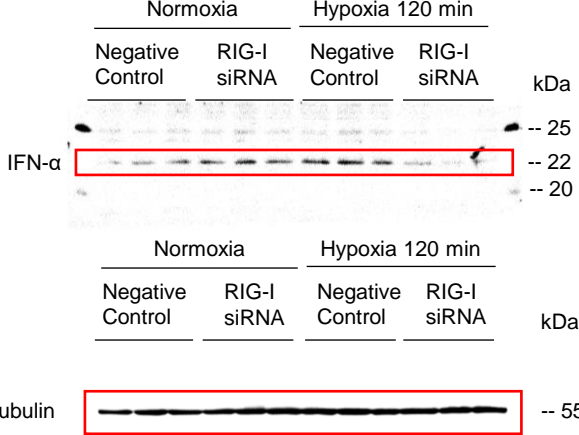

Fig 2C

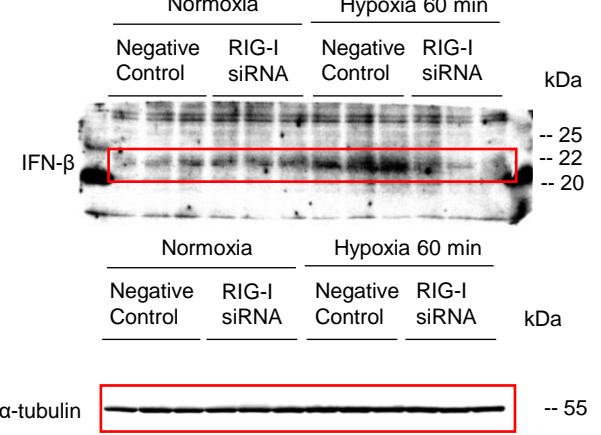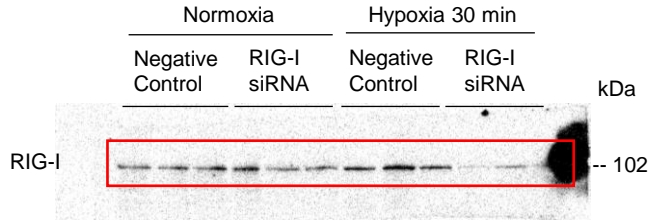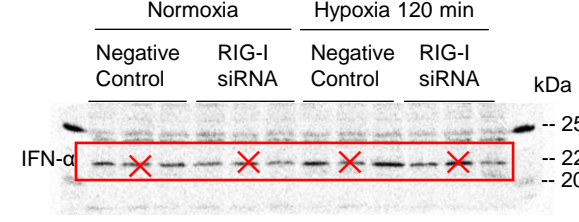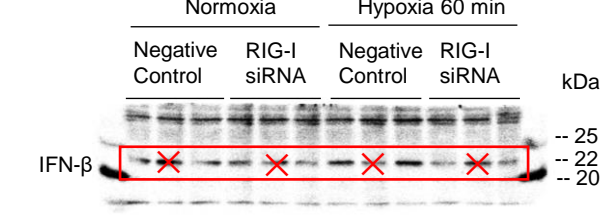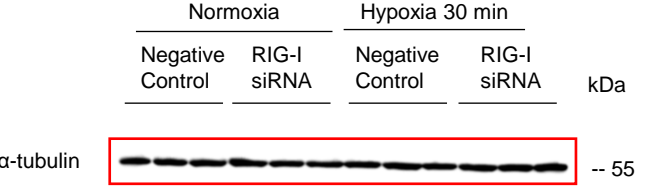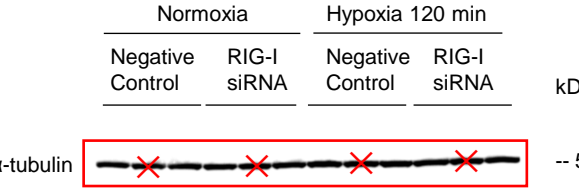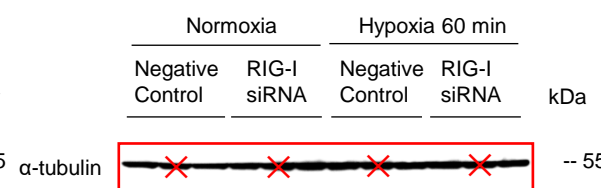

Supplement: S6 Fig — (PDF) [file pone.0258856.s006.pdf]

# S8 Fig. raw images of western blots for Fig 4A, 5A, 6A, and 7A.

Fig 4A

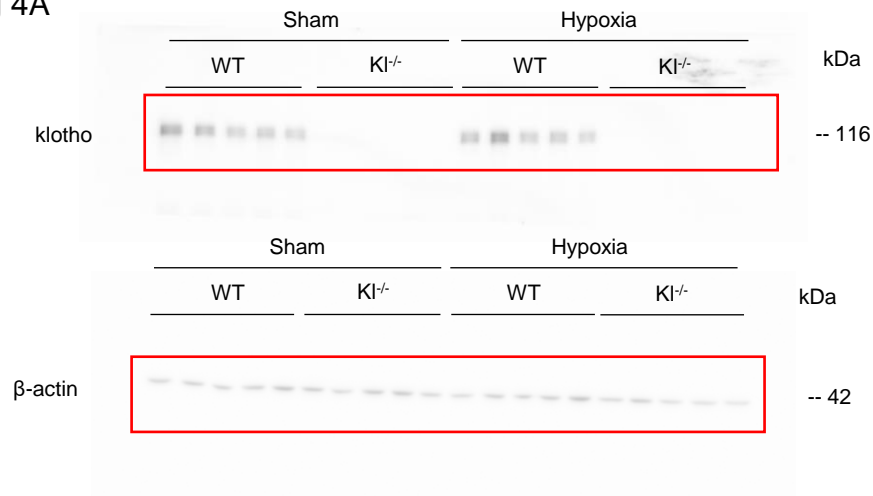

Fig 5A

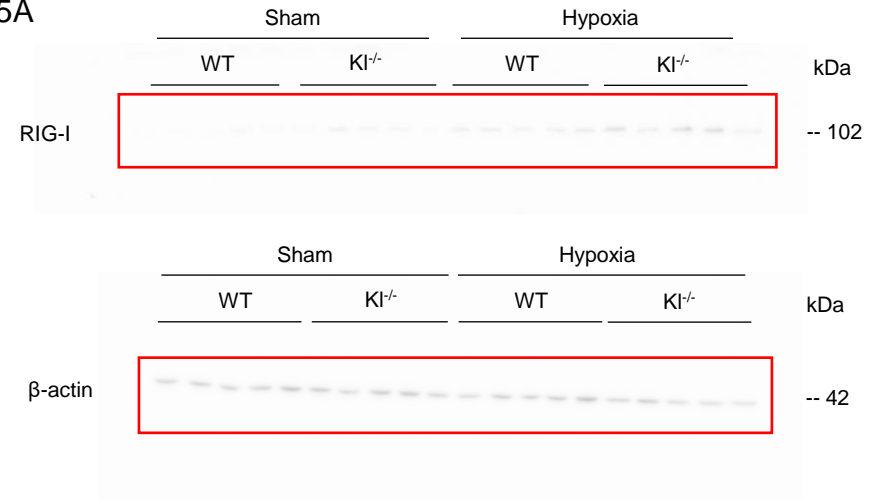

Fig 6A

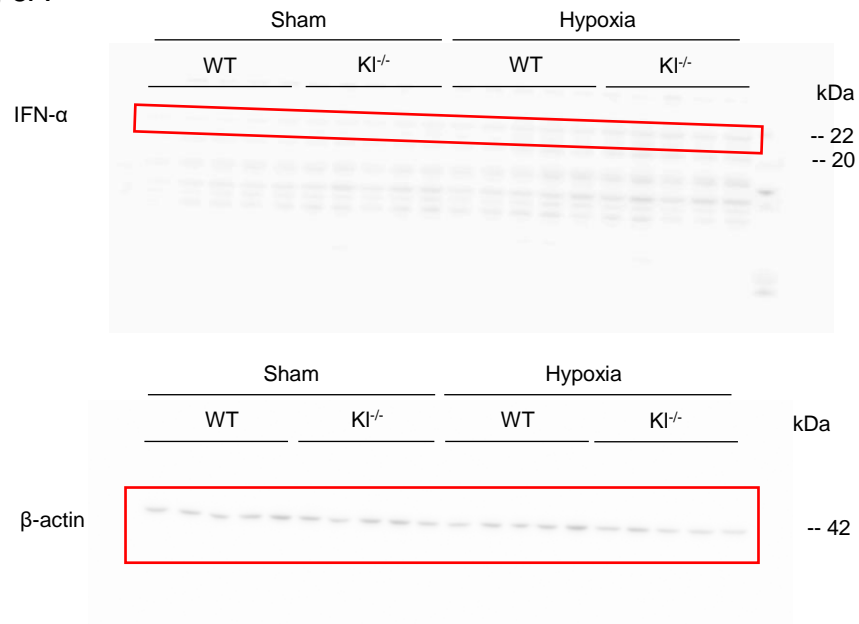

Fig 7A

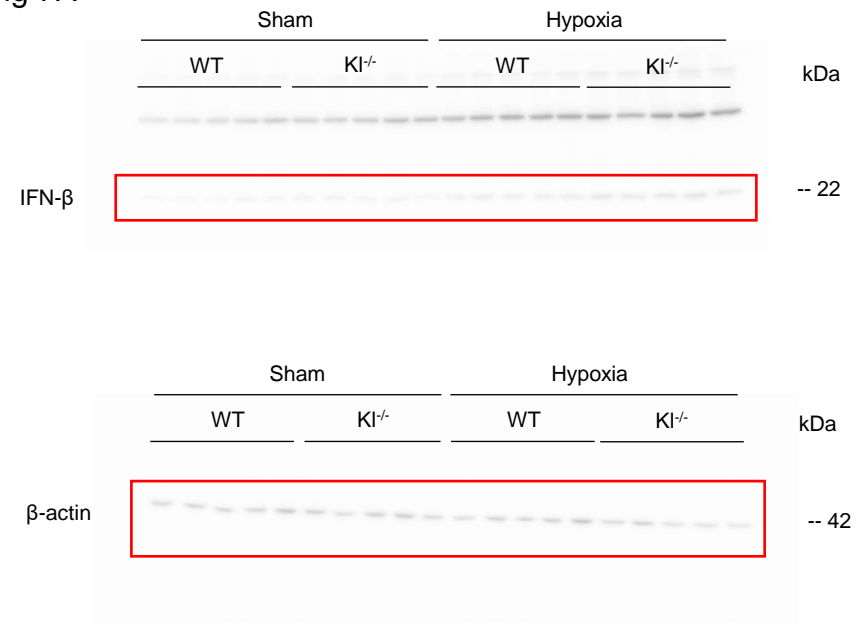

Supplement: S7 Fig — (PDF) [file pone.0258856.s007.pdf]

# S9 Fig. raw images of western blots for S1 Fig and S2 Fig.

## S1 Fig.

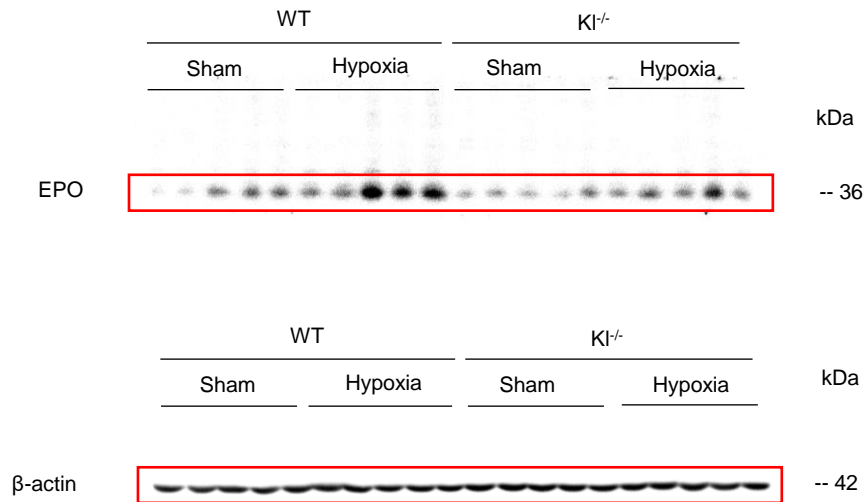

## S2 Fig.

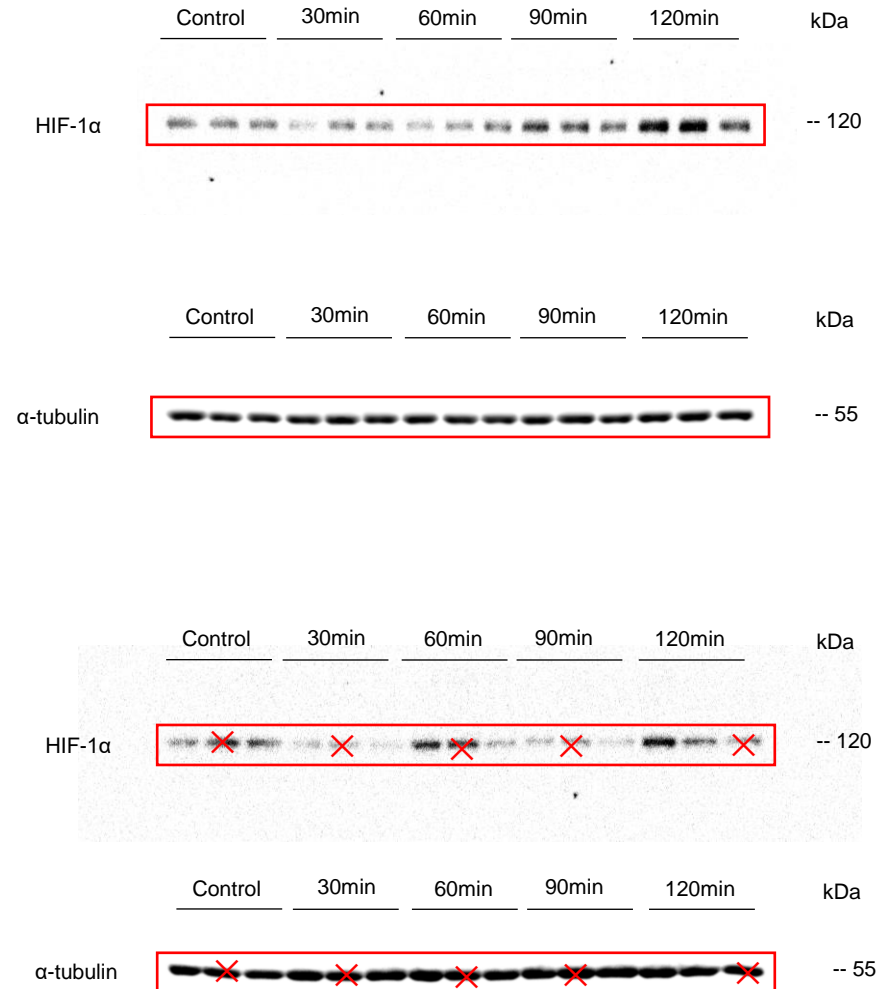

Supplement: S8 Fig — (PDF) [file pone.0258856.s008.pdf]
