# Supplementary material for: Klotho deficiency intensifies hypoxia-induced expression of IFN-α/β through upregulation of RIG-I in kidneys
Source: PLoS One. 2021 Oct 21;16(10):e0258856. doi: 10.1371/journal.pone.0258856 (PMC8530307; doi:10.1371/journal.pone.0258856)
Supplement: S1 Table — (PDF) [file pone.0258856.s009.pdf]

S3 Table. Clinical characteristics related to renal function of IgA nephropathy patients

Variables	Total (n = 33)
Age	45.9 ± 11.6
Male sex	51.5%
Body mass index	24.2 ± 4.2
Renin-angiotensin system inhibitor	24.2%
Systolic blood pressure (mmHg)	127.5 ± 14.7
Diastolic blood pressure (mmHg)	77.8 ± 9.8
Total urinary protein (g/day)	1.08 ± 1.62
Cr (mg/dL)	1.11 ± 0.78
eGFR (mL/min/1.73 m^2)	62.7 ± 21.1
Creatinine clearance (mL/min)	89.9 ± 31.7
Total cholesterol (mg/dL)	209 ± 52.2
Triglyceride (mg/dL)	157 ± 111
LDL cholesterol (mg/dL)	123 ± 36.7
HDL cholesterol (mg/dL)	60.1 ± 15.8
Diabetes mellitus	6.2%
Hypertension	15.2%
Dyslipidemia	6.1%
Hyperuricemia	6.1%
The Oxford classification	
Mesangial hypercellularity	M1 : 24.2%
Segmental glomerulosclerosis	S1 : 45.5%
Endocapillary hypercellularity	E1 : 6.1%
Tubular atrophy / interstitial fibrosis	T1 : 18.2%, T2 : 0%

Abbreviations

eGFR : estimated glomerular filtration rate

The Japanese GFR equation based on serum creatinine (Cr) was used for eGFR.

$\text{eGFR (mL/min/1.73 m}^2) = 194 \times \text{Cr}^{-1.094} \times \text{Age}^{-0.287}$ (× 0.739 if female)

Cr : Creatinine

LDL : low-density lipoprotein, HDL : high-density lipoprotein
