# Supplementary material for: Klotho deficiency intensifies hypoxia-induced expression of IFN-α/β through upregulation of RIG-I in kidneys
Source: PLoS One. 2021 Oct 21;16(10):e0258856. doi: 10.1371/journal.pone.0258856 (PMC8530307; doi:10.1371/journal.pone.0258856)
Supplement: Attachment — Submitted filename: renamed_e4e28.docx [file pone.0258856.s010.docx]

Thank you for pointing out some issues with our manuscript. We have revised the manuscript in accordance with your comments. Our responses to each comment are shown below:

1.Please amend your Methods section to state the method of euthanasia/sacrifice of the rats.

Response to comment 1

The method of euthanasia/sacrifice was cardiac puncture. Following your suggestion, we have added this to the Methods section.

2.We now require authors to provide the original unadjusted and uncropped	images for any blot or gel data reported in PLOS ONE submissions. In our internal	checks for your submission, we noted that you did not provide original raw image files supporting blot/gel data in response to our previous request.

Please provide the unadjusted and uncropped images underlying all blot and gel figures at this time; see the above URL for instructions on how the raw blot/gel image data should be prepared and submitted.

Response to comment 2

Following your suggestion, we have provided the original unadjusted and uncropped images in the Supplemental Information.

3.We noticed you have some minor occurrence of overlapping text with the following previous publication(s), which needs to be addressed:

In your revision ensure you cite all your sources (including your own works), and quote or rephrase any duplicated text outside the methods section. Further consideration is dependent on these concerns being addressed.

Response to comment 3

As you have pointed out, some of the Methods section overlapped with our previous publication. Following your suggestion, we have rewritten the Methods section to resolve this.
